# Comparison of two methods of laparoscopic trocar insertion (Hasson and Visiport) in terms of speed and complication in urologic surgery

**DOI:** 10.1051/bmdcn/2018080422

**Published:** 2018-11-26

**Authors:** Mehrdad Mohammadi, Behnam Shakiba, Matin Shirani

**Affiliations:** 1 Department of Urology, Isfahan University of Medical Sciences Isfahan Iran

**Keywords:** Complication, Laparoscopy, Urological surgical procedure

## Abstract

Background: Nowadays, diverse approaches have been existed for laparoscopic procedures. The most common laparoscopic entry methods included close and direct entry laparoscopy and open (Hasson) laparoscopy. There is no evidence regarding the superiority in safety and initial speed for the use of open and optical laparoscopic entry. Therefore, the sight of current study was to evaluate comparative survey of two methods of laparoscopic trocar insertion (Hasson and VisiportTM) in terms of speed and complications in urologic surgery.

Methods: This expertized base clinical trial study was conducted on 100 patients who underwent urological laparoscopy in Alzahra Hospital, Isfahan, Iran. These patients were randomly divided to two groups (n = 50). One group underwent open laparoscopy and another group Visiport optical trocar. Speed and Complications of urologic surgery was extracted from medical records. Independent *T* test was used for doing of analysis.

Results: The mean age of patients in Hasson and Visiport laparoscopic group was 41.4 ± 11.2 and 41.6 ± 15 years old, respectively *(p* = 0.91). The mean time for initial trocar placement in patients who underwent Visiport trocar system and Hasson laparoscopic technique was 37.7 ± 15.59 and 95.4 ± 31.75 seconds. There was gratifying difference between two techniques of laparoscopic trocar insertion (Hasson and Visiport) in terms of speed *(p* = 0.000). In addition, complications were observed in 8% of patients who underwent Visiport trocar system. However, no complications were observed in Hasson laparoscopy group.

Conclusion: Visiport optical trocar technique is faster for initial trocar placement than open laparoscopy. However it is associated with complications compared to open laparoscopy. Therefore, there is evidence of benefit in terms of speed for initial trocar placement and harm based on complications in Visiport trocar system.

## 1. Introduction

In Laparoscopy technique, we evaluate the abdominal cavity [1–10]. It needs insertion of a cannula to visualize the abdomen’s cavity with an illuminated telescope [11]. Annually, more than 2 million laparoscopy techniques are performed in the United States, containing a vast span of general, urologic and gynecologic surgical methods [12]. Urological laparoscopy can be used in diagnostic surgeries and reconstructive operations. Moreover, laparoscopy can increasingly use for a variety of urological methods [13].

A significant alteration was observed in laparoscopic methods in the United Kingdom and international places [14–18]. It also has diverse benefits such as lower coherence risk and faster recovery time after hospitalization. However it is associated with complications during entry to abdomen including visceral injury, urological tract injury, hemorrhage, herniation and infection [19]. Main and major complications happen during entry of laparoscopy to abdominal wall. Such complications may cause mortality.

Moreover, another parameter for performing of laparoscopy is significance of time. The importance of time for performing of laparoscopy is that with reducing of entry time, overall time of surgery is decreased and complication of anesthesia and general surgery is also reduced. Nowadays, diverse approaches have been created for laparoscopic technique [20, 21]. Several international bodies have proposed principal of safe laparoscopic entry [22–27]. The most commonly laparoscopic entry methods which have been principally used in general surgery included close and direct entry laparoscopy and open (Hasson) laparoscopy [20]. Superiority of open entry is due to low probability of vascular injury [28]. Moreover, this opinion also is associated with some challenges. To overpass these complications, optically guided trocars are suggested for dropping the injury risk to intra-abdominal construction by permitting the surgeon to observe abdominal structures in placement [28].

The Visiport (a kind of optical trocars) is a disposable and expendable visual entry tool which includes a cannula and hollow trocar [11]. It is applied after insufflation of CO2. This technique is palmed *via* surgeon’s hand and maintained perpendicular to distend patient’s CO2 to abdomen [11]. When accurate anatomical status of trocar tip is checked by monitor, downward axial pressure is used and activated trigger. Downward pressure causes trocar tip situation is checked again. These sequences are repeated till the peritoneal cavity is arrived. This is not fired till the accurate anatomical status of trocar tip is known. However, none of the laparoscopic entry methods have distinct superiority over other. On the other words, all of these techniques are associated with abundant complication [29]. This has caused main alteration in laparoscopic entry practice [30–32] among clinicians at international locations to select kind of entry method [20].

Since, there is no evidence regarding safety and initial rate for the use of laparoscopic trocar insertion such as Hasson and Visiport, we compare two methods of laparoscopic trocar insertion (Hasson and Visiport) in terms of speed and complication in urologic surgery.

## 2. Materials and Methods

### 2.1 Electing of population

This expertized base clinical trial study was conducted on 100 patients who underwent urological laparoscopy in Alzahra Hospital, Isfahan, Iran in 2017. After taking consent from patients, this study was approved by Isfahan University of Medical Sciences. The number which was assigned in ethical committee to current manuscript was 396088. These patients were randomly divided to two groups (n = 50). One group underwent open laparoscopic treatment and another group Visiport trocar system.

### 2.2 Inclusion and exclusion criteria

Inclusion criteria were the following: over the age of 18 years and candidates for urological laparoscopic surgery. Moreover, complications including uncorrected coagulopathy, Ileus, infection of the abdominal wall, history of open abdominal surgery, presence of malignant ascites and generalized peritonitis caused to exclude patients from study.

### 2.3 Data collection

Data including demographic characteristics, laparoscopic entry and the most common possible complications of this process including the amount of consumption CO2, vessels damage, subcutaneous emphysema and Trocar’s infection were extracted from medical records.

### 2.4 Statistical analysis

We applied SPSS version 19. Independent *T* test was used for doing of analysis. Statistically, P-value < 0.05 was advised.

## 3. Results

### 3.1 Demographic features of current population

In current study, 51 (51%) and 49 (49%) patients were male and female, respectively. Therefore, two groups almost were homologous. In addition, the mean age of patients in two groups (open and Hasson) was 41.4 ± 11.2 and 41.6 ± 15, respectively (*p* = 0.91).

### 3.2 Comparison of two methods of laparoscopic trocar insertion (Hasson and Visiport) in terms of speed

Table 1 shows comparison of two methods of laparoscopic trocar insertion (Hasson and Visiport) in terms of mean time. As shown in Table 1, the mean time at the initial entry in patients who underwent Visiport laparoscopic visual trocar was lower than *patients* who underwent open laparoscopic treatment. It shows that Visiport laparoscopic trocar is performed faster than open laparoscopy. There was gratifying difference between two techniques of laparoscopic trocar insertion (Hasson and Visiport) in terms of speed of the initial entry (*p* = 0.000). The most common type of surgery in *patients* who underwent Visiport *laparoscopic visual trocar* was pyeloplasty (26%) and then nephrectomy (22%) and the least common type was kidney stone (2%). The most common type of surgery in *patients* who underwent Open laparoscopic treatment was nephrectomy (42%) and the least was pelvic stone (2%).

**Table 1:** Comparison of two methods of laparoscopic trocar insertion (Hasson and Visiport) in terms of speed.

**Mean Time (Second)**		
Visiport laparoscopic visual trocar (group 1)	Open laparoscopy (group 2)	*p*-value

37.7 ± 15.59	95.4 ± 31.75	0.000

### 3.3 Complication of Visiport trocar system

Complication of Visiport trocar system is shown in Table 2. As shown in Table 2, complications were observed in 8% of these patients. In addition, no complications were observed in Hasson laparoscopy group.

**Table 2:** Complication of Visiport trocar system.

**Complication of Visiport trocar system**	**Number (Percent)**

Subcutaneous emphysema	1(2%)
Trocar site infection	1(2%)
Mild Liver damage	1(2%)
Small vessel damage	1(2%)
Total	4(8%)

## 4. Discussion

Laparoscopy has proved as a confident surgical procedure with abundant complications [33]. In our study, complications of Visi- port trocar system included subcutaneous emphysema, trocar site infection, mild liver damage and small vessel damage which were observed in 8% of patients, while, no complications were observed in Hasson laparoscopy group. Moreover, in current study, Visiport laparoscopy was performed faster than Hasson laparoscopy. It is noticed that the location and technique of initial entry into the abdominal wall (based on Visiport or Hadsson method) were completely similar and did not correlate with the type of surgery. Therefore, the difference in the type of surgery in the two groups did not affect our study.

Possible reasons of main complication are inappropriate placement of a Veress needle insertion or trocar which is resulted to vascular or bowel injury [33]. So that vascular injury is an original reason of death. Moreover, more than 50% of side effects of primary laparoscopy happen during entry to abdomen. The importance of time in the first trocar insertion is that by reducing the time of entry, the overall time of surgery decreases. Therefore, the complications of anesthesia and the complications of the surgery are reduced.

Thomas *et al,* demonstrated that optical access trocars are secure and quick technique for initial trocar placement. Moreover, these findings showed that optical access trocar is associated with few complications [33]. Other studies have shown that bowel injuries (30% to 50%) and vascular injuries (13% to 50%) are not diagnosed in surgery time [27]. Bowel injury compared to vascular injury is more common and produce serious outcome due to delay in diagnosis [11]. Another study compared closed and open entry techniques and reported that complication rate of closed and open technique was 0.07% and 0.17%, respectively [35]. They concluded that complications of open entry method were higher than closed entry method [36].

Vilos *et al.,* in 2007 reported that in open laparoscopy, the rate of infection, bowel and vascular injury was 0.4%, 0.1% and 0%, respectively and this rate in closed laparoscopy was 1%, 0.2%, and 0.2%, respectively. Chapron *et al.,* in 2003 reported that the major vessel injury rates in the closed and open technique were 0.01% and 0% in the open technique [37]. A meta-analysis reported that the incidence of vascular injury rate in closed laparoscopy was 0.44% compared with 0% in open laparoscopy [38]. Diverse studies showed advantage and complications of different laparoscopic entry methods [27].

Gunenj *et al.,* in 2005 obtained same results and demonstrated that direct trocar insertion is an easy, safe, and effective technique [39]. Tinelli *et al.,* in 2009 reported that optically guided trocars can reduce the danger of injury to abdominal construction which cause surgeon to observe abdominal wall layers in placement [28]. While, Sharp *et al.*, reported that optical-access trocars may cause main injuries despite having the capability to see tissue layers in period of insertion [34]. Another study compared direct optical access and Hasson methods and reported that visual entry system is associated with the increase of peace and safety, the decrease of time and the blood loss allowing visually guided entry.

Tinelli *et al.*, in another study compared direct optical entry (DOE) with classical open laparoscopy in patients underwent abdominal pelvic surgery. They concluded that direct optical entry is secure such as open laparoscopy [40]. However, Jansen reported that there is no document to superiority of closed entry method. Therefore, open or other procedure is still suggested [17]. Rai *et al.,* in another study reported that no benefit was observed between particular techniques in terms of safety [41]. Schoon derwoerd *et al.,* in 2005 reported that for diminishing the risk of peritoneal entry, open-access technique like Hasson trocar is preferred than other technique including radially expanding trocars and direct trocar [42]. Moreover, they reported that no difference was observed between these techniques for arresting visceral and vascular complications. It seems that optical trocars combine the benefits of the diverse entry techniques and prepare a sure and possible insertion method of laparoscopy for patients. Molloy *et al.,* reported that open entry is also associated with bowel injury. It seems that direct entry may be safe alternative than open entry technique [43].

In addition, serious heterogeneity in techniques such as laparoscopic entry practice was due to the attendance of risk factors in entry method [20]. Krishnakumar *et al.,* reported that open procedures are commonly used for high-risk patients, like those with a previous abdominal surgery, pregnant women, children and *etc* [2]. Perhaps, this is the cause of more complications in open method.

## 5. Conclusion

According to results of this study, Visiport optical trocar technique is faster for initial trocar placement than Hadsson laparoscopy. However, it is associated with complications compared to open laparoscopy. Therefore, there is benefit with respect to speed for initial trocar placement and harm based on complications in Visiport trocar system.

## Conflict of interest

The authors declare that there is no conflict of interest.

## References

[1] Corson SL , Chandler JG , Way LW . Survey of laparoscopic entry injuries provoking litigation. J Am Assoc Gynecol Laparosc. 2001; 8: 341–7. 11509771 10.1016/s1074-3804(05)60328-3

[2] S. Krishnakumar , P. Tambe . Entry complications in laparoscopic surgery. JGES. 2009; 1(1): 4–11. 22442503 10.4103/0974-1216.51902 PMC3304260

[3] C. Nezhat , J. Cho , V. Morozov , P. Yeung . Preoperative periumbilical ultrasound-guided saline infusion (PUGSI) as a tool in predicting obliterating subumbilical adhesions in laparoscopy. Fertility and Sterility. 2009; 91(6): 2714–9. 18565517 10.1016/j.fertnstert.2008.03.073

[4] Rohatgi , A. L. Widdison . Left subcostal closed (Veress needle) approach is a safe method for creating a pneumoperitoneum. J Lap- aroendosc Adv Surg Tech. 2004; 14(5): 278–80. 10.1089/lap.2004.14.278 15630943

[5] Y. Afifi , A. Raza , M. Balogun , K. S. Khan , R. Holder . New nomogram for safe laparoscopic entry to reduce vascular injury. J Obstet Gynaecol. 2011; 31(1): 69–72. 21280998 10.3109/01443615.2010.529517

[6] Hypólito OH , Azevedo JL , Caldeira De Lima Alvarenga FM , De Azevedo OC , Miyahira SA , Miguel GP , et al Creation of pneumoperitoneum: noninvasive monitoring of clinical effects of elevated intraperitoneal pressure for the insertion of the first troca. Surgical Endoscopy and Other Interventional. Techniques. 2010; 24(7): 1663–9. 10.1007/s00464-009-0827-2 20035347

[7] Azevedo JL, Azevedo OC, Miyahira SA, Miguel GP, Becker OM Jr, Hypolito OH, et al Injuries caused by Veress needle insertion for creation of pneumoperitoneum: a systematic literature review,” Surgical Endoscopy and Other Interventional Technique 2009; 23(7): 1428–1432, 2009. 10.1007/s00464-009-0383-9 19263124

[8] O. C. de Azevedo , J. L. M. C. Azevedo , A. A. Sorbello , G. P. S. Miguel , J. L. Wilson , A. C. De Godoy . Evaluation of tests performed to confirm the position of the Veress needle for creation of pneumoperitoneum in selected patients: a prospective clinical trial. Acta Cirurgica Brasileira. 2006; 21(6): 385–91. 17160250 10.1590/s0102-86502006000600006

[9] Merlin T , Hiller J , Maddern G , Jamieson GG , Brown AR , Kolbe A . Systematic review of the safety and effectiveness of methods used to establish pneumoperitoneum in laparoscopic surgery. Br J Surg. 2003; 90: 668–70. 12808613 10.1002/bjs.4203

[10] Munro MG . Laparoscopic access: complications, technologies and techniques. Curr Opin Obstet Gynecol. 2002; 14: 365–74. 12151825 10.1097/00001703-200208000-00002

[11] Vilos G. A. . Laparoscopic Entry: A Review of Techniques, Technologies, and Complications. SOGC clinical practice guidline. J ObstetGynaecol Can. 2007; 29(5): 433–47. 10.1016/S1701-2163(16)35496-2 17493376

[12] Fuller J, MPH D, Ashar B. Trocar-associated injuries and fatalities: An analysis of 1399 reports to the FDA. J Minimally Invasive Gynecol. 2005; 12. 10.1016/j.jmig.2005.05.008 16036187

[13] Paulter S . Assesment of risk for inra- abdomen adhesions as laparoscopy for urological tumors. J Urol 2002; 7: 1–10. 10.1016/S0022-5347(05)64152-3 12441924

[14] Verma R . Laparoscopic entry techniques: clinical guideline, national survey, and medicolegal ramifications. Surg Endosc. 2008; 22: 2686–97. 18401653 10.1007/s00464-008-9871-6

[15] Ahmad G , Duffy JMN , Watson AJS . Laparoscopic entry techniques and complications. Int J Gynaecol Obstet. 2007; 99(1): 52–5. 17628561 10.1016/j.ijgo.2007.04.042

[16] Chin K , Newton J . Survey of training in minimal access surgery in the West Midlands region of the UK. Gynacol Endosc 1996; 5(6): 329–333.

[17] Lalchandani S , Philips K . Laparoscopic entry technique-a survey of practices of consultant gynaecologists. Gynecol Surg. 2005; 2(4): 245–9.

[18] Lingam K , Cole RA . Laparoscopic entry port visited: a survey of practices of consultant gynaecologists in Scotland. Gynaecol Endosc 2001; 10(5): 335–42.

[19] Cuss A, B M, Bhatt M. Coming to TermsWith the Fact That the Evidence for Laparoscopic Entry Is as Good as It Gets. JMIG. 2014; 10: 23–30. 10.1016/j.jmig.2014.10.023 25460522

[20] Varma R , Gupta J . Laparoscopic entry techniques: clinical guideline, national survey, and medicolegal ramifications. SurgEndosc. 2008; 22: 2686–97. 10.1007/s00464-008-9871-6 18401653

[21] Abdelmaksoud AAAA , Biyani Ch . Laparoscopic approaches in urology. B J U. 2005; 95: 244–9. 10.1111/j.1464-410X.2005.05277.x 15667648

[22] Neudecker J , Sauerland S , Neugebauer E , Bergamaschi R , Bonjer HJ , Cuschieri A , et al (EAES) The European Association for Endoscopic Surgery clinical practice guideline on the pneumoperitoneum for laparoscopic surgery. Surg Endosc. 2002; 16(7): 1121–43. 12015619 10.1007/s00464-001-9166-7

[23] SAGES. Society of American Gastrointestinal Endoscopic Surgeons (SAGES). SAGES guidelines for diagnostic laparoscopy. Los Angeles (CA): Society of American Gastrointestinal Endoscopic Surgeons (SAGES); 2002; 1: 2–9.

[24] Pierre F , Chapron C , Deshayes M , Madelenat P , Magnin G , Quer- leu D . Initial access for laparoscopic gynecologic surgery. French Society of Endoscopic Gynecology, International Society of Pelvic Surgery and the National College of French Gynecologists-Obstetri- cians. J Gynecol Obstet Biol Reprod (Paris) 2000; 29(1): 8–12. 10675828

[25] Bakkum EA , Timmermans A , Admiraal JF , Brolmann HAM , Jansen FW . Laparoscopic entry techniques: a protocol for daily gynaecological practice in The Netherlands. Gynecol Surg. 2006; 3(2): 84–7.

[26] Garry R . Laparoscopic surgery. Best Pract Res Clin Obstet Gynaecol. 2006; 20(1): 89–104. 16373090 10.1016/j.bpobgyn.2005.10.003

[27] Vilos GA . (2006) The ABCs of a safer laparoscopic entry. J Minim Invasive Gynecol 2006; 13(3): 249–51. 16698536 10.1016/j.jmig.2005.12.005

[28] Tinelli A . Abdominal Access in Gynaecologic Laparoscopy: A Comparison Between Direct Optical and Open Access. J Laparoen- doscAdvSurg Tech A. 2009; 19(4): 1–4. 10.1089/lap.2008.0322 19397397

[29] WongW Felix . A safe optically guided entry technique using Endo- path Xcel Trocars in laparoscopic surgery: A personal series of 821 patients. GMIT. 2013; 2: 30e33-30e38.

[30] Jansen FW , Kolkman W , Bakkum EA , de Kroon CD . Kroon CD, Trimbos-Kemper TC, Trimbos JB. Complications of laparoscopy:an inquiry about closed-versus open-entry technique. Am J ObstetGynecol. 2004; 190(3): 634–8. 10.1016/j.ajog.2003.09.035 15041992

[31] Kaloo P , Cooper M , Molloy D . A survey of entry techniquesand complications of members of the Australian Gynaecological Endoscopy Society. Aust N Z J ObstetGynaecol. 2002; 42(3): 264–6. 10.1111/j.0004-8666.2002.00264.x 12230060

[32] Marret H , Golfier F , Cassignol A , Raudrant D . Methods for laparoscopy: open laparoscopy or closed laparoscopy?Attitude of the French Central University Hospital. Gynecol Obstet Fertil. 2001; 29(10): 673–67. 11732433 10.1016/s1297-9589(01)00206-5

[33] Thomas M . Optical access trocar injuries in urological laparoscopic surgery. J Urol. 2003; 170: 61–3. 12796645 10.1097/01.ju.0000067622.28886.75

[34] Sharp HT , Dodson MK , Draper ML , Watts DA , Doucette RC , Hurd WW . Complications associated with optical-access laparoscopic trocars. Obstet Gynecol. 2002; 99(4): 553–5. 12039109 10.1016/s0029-7844(02)01656-3

[35] F. W. Jansen , W. Kolkman , E. A. Bakkum , C. D. De Kroon , T. C. M. Trimbos-Kemper , J. B. Trimbos . Complications of laparoscopy: an inquiry about closed- versus open-entry technique. American J Obstetrics Gynecol. 2004; 190(2): 3634–8. 10.1016/j.ajog.2003.09.035 15041992

[36] Toro A, Maurizi M, Giovanni Cappello G. Comparison of Two EntryMethods for Laparoscopic Port Entry: Technical Point of View. Diagn Ther Endosc. 2012; 1–7. 10.1155/2012/305428 PMC3384909 22761542

[37] C. Chapron , L. Cravello , N. Chopin , G. Kreiker , B. Blanc , J. B. Dubuisson . Complications during set-up procedures for laparoscopy in gynecology: open laparoscopy does not reduce the risk of major complications. Acta Obstetricia et Gynecologica Scandinavica. 2003; 82(12): 1125–9. 14616258 10.1046/j.1600-0412.2003.00251.x

[38] Larobina M , Nottle P . Complete evidence regarding major vascular injuries during laparoscopic access. Surg Laparosc Endosc Percutan Tech. 2005; 15(3): 119–23. 15956893 10.1097/01.sle.0000166967.49274.ca

[39] Günen§ MZ, Yesildaglar N, Bingol B, Onalan G, Tabak S, Gokmen B. The safety and efficacy of direct trocar insertion with elevation of the rectus sheath instead of the skin for pneumoperitoneum. Surg Laparosc Endosc Percutan Tech. 2005; 15(2): 80–1. 15821619 10.1097/01.sle.0000162106.95875.b9

[40] Tinelli Al, Malvasi A, Guido M, Tsin DA, Hudelist G, Stark M, et al Laparoscopy entry in patients with previous abdominal and pelvic surgery. Surgical Innovation. 2011; 18(3): 201–5. 21245070 10.1177/1553350610393989

[41] Rai M . Comparison between different entry technique in performing laparoscopic gynecological surgeries. World Journal of Laparoscopic surgery. 2015; 8(3): 101–9.

[42] Schoonderwoerd L , Swank DJ . The role of optical access trocars in laparoscopic surgery. SurgTechnol Int. 2005; 14: 61–7. 16525956

[43] Molloy D , Kaloo PD , Cooper M , Nguyen TV . Laparoscopic entry: a literature review and analysis of techniques and complications of primary port entry. Aust N Z J Obstet Gynaecol. 2002; 42(3): 246–54. 12230057 10.1111/j.0004-8666.2002.00246.x

